# Hypoxia-Induced ROS Contribute to Myoblast Pyroptosis during Obstructive Sleep Apnea via the NF-*κ*B/HIF-1*α* Signaling Pathway

**DOI:** 10.1155/2019/4596368

**Published:** 2019-12-11

**Authors:** Li-Ming Yu, Wei-Hua Zhang, Xin-Xin Han, Yuan-Yuan Li, Yun Lu, Jie Pan, Jia-Qi Mao, Lu-Ying Zhu, Jia-Jia Deng, Wei Huang, Yue-Hua Liu

**Affiliations:** ^1^Department of Orthodontics, Shanghai Stomatological Hospital, Fudan University, Shanghai 200001, China; ^2^Oral Biomedical Engineering Laboratory, Shanghai Stomatological Hospital, Fudan University, Shanghai 200001, China; ^3^Department of endodontics, Stomatological Hospital, Hebei Medical University, Shijiazhuang 050017, China; ^4^Xiangya School of Stomatology, Xiangya Stomatological Hospital, Central South University, Changsha 410000, China

## Abstract

Tissue hypoxia caused by upper airway collapse is a main cause of excessive oxidative stress and systemic inflammation in obstructive sleep apnea (OSA) patients. Increased reactive oxygen species (ROS) and inflammatory responses affect cell survival and ultimately contribute to tissue injury. In the present study, we proposed that the induction of ROS by hypoxia, as an intrinsic stress, activates myoblast pyroptosis in OSA. We found increased cell death and abnormal expression of pyroptosis markers in the skeletal muscle of OSA mice. In vitro studies showed hypoxia-induced pyroptotic death of C2C12 myoblasts, as evidenced by the activation of caspase-1 and gasdermin D (GSDMD). Hypoxia induced ROS overproduction and accumulation in myoblasts. More importantly, applying N-acetylcysteine (NAC), an ROS scavenger, rescued cell swelling, downregulated the inflammatory response, and prevented pyroptotic death in hypoxia-cultured myoblasts. Hypoxia stimulation promoted NF-*κ*B P65 phosphorylation and HIF-1*α* nuclear translocation. Moreover, hypoxia increased the nuclear level of cleaved caspase-1 and GSDMD. NAC inhibited hypoxia-induced variations in the HIF-1*α* and NF-*κ*B signaling pathway. Taken together, our results determined that hypoxia-induced ROS contribute to myoblast pyroptosis. Therefore, our findings suggest that ROS may be a potential therapeutic target for ameliorating hypoxia-induced cell death and tissue injury, especially in OSA and hypoxia-related diseases.

## 1. Introduction

Obstructive sleep apnea (OSA) is a globally prevalent disorder that is characterized by snoring, fragmented sleep, and decreased oxygen saturation. Approximately 17% of men and 9% of women aged 50-70 years suffer from this disorder [1], and its prevalence is higher in the obese population [2]. The key feature of OSA is hypoxia, which results from upper airway obstruction during sleep. Chronic hypoxia impairs tissue homeostasis and is closely associated with comorbidities such as metabolic dysfunction, cardiovascular diseases, and cognitive decline [3]. In addition, OSA is associated with sexual dysfunction and increased motor vehicle accidents [2, 4].

Reactive oxygen species (ROS) are produced during a variety of cellular processes. Studies have shown increased ROS levels and oxidative stress in OSA patients [5]. Our previous study also found increased oxidative stress in the skeletal muscle of OSA rats [6]. Under normal conditions, skeletal muscle produces ROS at a low level that increases during muscle fiber contraction. An insufficient oxygen supply results in excessive ROS production and accumulation [7]. Increased oxidative stress impairs muscle function and structure. Muscle-specific stem cells are satellite cells in a quiescent state. Upon stress and injury, these cells are activated and proliferate into myoblasts for tissue repair. However, hypoxia inhibits the differentiation of myoblasts [8]. In addition, hypoxia induces oxidative injury and apoptosis in genioglossus myoblasts [9].

Cell death is a crucial and essential process in maintaining tissue homeostasis. It occurs in response to diverse triggers, especially oxidative stress [10]. According to an update by the Nomenclature Committee on Cell Death (NCCD), regulated cell death (RCD) can be divided into several categories, including apoptosis, autophagy, pyroptosis, and ferroptosis [11]. A recent study reported increased levels of cell death biomarkers in the sera of patients with OSA [12]. Hypoxia-induced apoptosis and autophagy have been reported in myoblasts [9, 13]. OSA can evoke apoptosis in the cardiac muscle [14]. Furthermore, accumulating evidence has demonstrated that autophagy is also involved in OSA [15]. However, it is unknown whether OSA can induce pyroptosis in skeletal myoblasts.

Pyroptosis, a specific type of nonapoptotic RCD, is characterized by inflammatory caspase activation and pore formation in the cell plasma membrane [11]. Due to extrinsic and intrinsic stimuli, inflammasomes are activated and cleave pro-caspase-1 into caspase-1. Then, caspase-1 mediates the cleavage of gasdermin D (GSDMD) and pro-IL-1*β* into the active forms. GSDMD is a key downstream effector in cell pyroptosis [16]. It forms pores in the plasma membrane that ultimately cause cell swelling and membrane lysis. Moreover, pyroptosis is a type of inflammatory cell death that is closely related to both infectious and noninfectious diseases [17]. ROS act as an intrinsic stimulus that triggers cell pyroptosis. Oxidative stress mediates pyroptosis in different cell types, including cardiomyocytes, macrophages, and neuronal cells [18–20]. However, the potential role of pyroptosis and its underlying signaling pathway in hypoxia-induced myoblasts is worthy of further investigation.

Hypoxia-inducible factor-1 alpha (HIF-1*α*) is the master transcription factor in response to cell hypoxia. In response to hypoxia, activated HIF-1*α* translocates to the nucleus and initiates target gene transcription. HIF-1*α* inhibition reduces cell death in renal tubular epithelial cells [21]. We previously reported that estradiol can improve the function of the upper airway muscle by inhibiting HIF-1*α* expression in OSA [22]. In addition, OSA patients also exhibit increased systemic inflammation. The nuclear factor-*κ*B (NF-*κ*B) family is a family of transcription factors that act as crucial regulators in inflammatory diseases. The NF-*κ*B cascade is upregulated in the fat tissue of OSA patients [23]. Moreover, crosstalk between HIF-1*α* and NF-*κ*B controls the response in a variety of medical conditions [24].

In this study, we found increased pyroptotic cell death in the skeletal muscle of OSA mice. Cobalt chloride- (CoCl_2_-) induced hypoxia activated inflammatory caspase-1 and the downstream effector GSDMD in C2C12 myoblasts. The inhibition of caspase-1 and GSDMD partly ameliorated C2C12 pyroptosis. Importantly, hypoxia significantly stimulated ROS generation and accumulation in C2C12 myoblasts, and applying an ROS scavenger (N-acetyl-L-cysteine (NAC)) protected myoblasts from hypoxia-induced pyroptotic injury. In addition, we found that NF-*κ*B P65 phosphorylation and HIF-1*α* nuclear translocation were involved in the response to hypoxia. Together, our findings demonstrate that cell pyroptosis plays an important role in the skeletal myoblasts of OSA mice, providing a novel and potential therapeutic target for OSA patients.

## 2. Materials and Methods

### 2.1. Animals and OSA Model

The study was approved by the Animal Welfare and Ethics Group, Department of Laboratory Animal Science at Fudan University, and all the animals were maintained and used in accordance with the Guide for Care and Use of Laboratory Animals. An OSA mouse model was prepared and created by our previously published procedures [25]. Briefly, C57BL/6J male mice (6-8 weeks old) were divided into 2 groups: control and OSA (*n* = 5). Intermittent hypoxia or normoxia air was supplied for 8 hrs per day. For OSA model, oxygen concentrations in mouse chambers were monitored by an O_2_ analyzer. During daytime, hypoxia and reoxygenation were manipulated by varying oxygen and nitrogen concentrations. The intermittent hypoxia cycles consisted of 2 minutes of hypoxia at 7 ± 1% O_2_ followed by reoxygenation at 21 ± 0.5% O_2_. For control mice, normoxia air was supplied. All mice were sacrificed after 5 weeks of OSA mimicking procedures.

### 2.2. TUNEL and Immunofluorescence Tissue Staining Protocols

Muscle samples were fixed in 4% paraformaldehyde at 4°C overnight. Then, they were conventionally prepared for paraffin embedding and sectioned into 4-*μ*m slides. For the TUNEL assay, a One-Step TUNEL Assay Kit (Beyotime, Shanghai, China) was used. For immunofluorescence staining, tissue sections were boiled in 10 mM citrate buffer (pH 6.0) for 10 minutes and then processed with 0.25% Triton-100 for 30 minutes. A primary antibody against NLRP3 (1 : 250) was purchased from Abcam (Cambridge, UK). A caspase-1 antibody (1 : 50) was purchased from Santa Cruz (TX, USA). The sections were incubated in primary antibodies at 4°C overnight. The secondary antibodies were Alexa Fluor 488-conjugated donkey anti-mouse and Alexa Fluor 594-conjugated donkey anti-rabbit antibodies and were purchased from Jackson ImmunoResearch (PA, USA). Then, the sections were stained with DAPI for 5 minutes in the dark, and micrographs were taken with a Leica DM2500 (Wetzlar, Germany) microscope.

### 2.3. C2C12 Culture and Hypoxia Treatment

C2C12 mouse myoblasts (Stem Cell Bank of Chinese Academy of Sciences, Shanghai, China) were cultured in high-glucose DMEM supplemented with 10% FBS (Gibco, MA, USA) and 1% penicillin/streptomycin in 5% CO_2_ at 37°C. Cobalt chloride (Sigma-Aldrich, MO, USA), also known as CoCl_2_, was dissolved in double-distilled water to generate a 20 mM stock solution after filtration through a 0.22-*μ*m PES membrane. CoCl_2_ was used to mimic hypoxic conditions in this study.

### 2.4. Lactate Dehydrogenase (LDH) Release Assays

Cytoplasmic LDH can be released into the medium following cell death. Cells were seeded in 96-well plates, and the culture supernatants were collected after centrifugation at 400 x g for 5 minutes. LDH levels were detected with a LDH release assay kit (Beyotime, Shanghai, China) following the manufacturer's recommendations. The absorbance values were determined at 495 nm by an Epoch 2 microplate spectrophotometer (Biotek, VT, USA).

### 2.5. Real-Time Polymerase Chain Reaction (PCR)

Cell total RNA was isolated using TRIzol (Life Technologies, CA, USA). Two micrograms of total RNA was used for reverse transcription with a FastQuant RT Kit (Tiangen, Beijing, China) according to the manufacturer's protocol. Then, real-time PCR was conducted by using SYBR Green Premix (Tiangen, Beijing, China) on a LightCycler 96 System (Roche, Basel, Switzerland). Differences in mRNA expression were determined by the comparative cycle threshold (Ct) value using 18S as the control. The sequences of the primers used for RT-PCR are presented in Table 1.

### 2.6. Western Blot Analyses

Cell samples were prepared in RIPA buffer or Laemmli buffer with inhibitors. For nuclear proteins, cell samples were collected and extracted following the instructions of the Nuclear and Cytoplasmic Protein Extraction Kit (Beyotime, Shanghai, China). Bis-tris or Tris-Gly gels were utilized to separate the protein samples after they were reduced and denatured. The proteins on the gels were transferred onto a PVDF membrane using a Bio-Rad transfer system. Antibodies against NLRP3 (1 : 1000) were purchased from Abcam (Cambridge, UK). Antibodies against GSDMD (1 : 100), IL-1*β* (1 : 100) and caspase-1 (1 : 100) were purchased from Santa Cruz (TX, USA). Antibodies against HIF-1*α* and caspase-1 (p20) were purchased from Novus Biologicals (CO, USA). Antibodies against total NF-*κ*B P65 (1 : 1000) and phosphorylated NF-*κ*B P65 (p-P65, 1 : 1000) were purchased from Cell Signaling (MA, USA). Antibody against actin (1 : 5000) was purchased from Absin (Shanghai, China). The secondary antibodies were purchased from Cell Signaling. The membranes were visualized using SuperSignal West Dura Substrate (Thermo Scientific, MA, USA), and the bands were detected with AI600 (GE Healthcare, IL, USA) and then quantified using the ImageJ program.

### 2.7. Cellular Reactive Oxygen Species (ROS)

The levels of cellular ROS were determined by a Reactive Oxygen Species Assay Kit (Beyotime, Shanghai, China) following the manufacturer's instructions. The nonfluorescent probe 2′, 7′-dichlorodihydrofluorescein diacetate (DCFH-DA) can be oxidized to DCF by cellular ROS. Briefly, C2C12 cells were incubated with DCFH-DA and Hoechst 33342 in DMEM at 37°C for 20 minutes. Then, the cells were washed with DMEM three times. The green fluorescence of DCF was visualized with a Leica DMi8 microscope, and the pictures were analyzed by ImageJ.

### 2.8. Caspase-1 Activity Assay

The caspase-1 enzyme level was measured with a Caspase-1 Activity Assay Kit (Beyotime, Shanghai, China) following the manufacturer's recommendations. Briefly, C2C12 cells were collected and lysed at 4°C. The supernatants were incubated with a substrate of caspase-1 (Ac-YVAD-pNA) to produce the yellow formazan product p-nitroaniline (pNA) at 37°C for 2 hours. The pNA levels were detected at 405 nm by an Epoch 2 microplate spectrophotometer (Biotek, VT, USA).

### 2.9. Enzyme-Linked Immunosorbent Assay (ELISA) for IL-1*β*

IL-1*β* levels in the supernatant of C2C12 cells were determined with a mouse IL-1*β* ELISA kit (BioLegend, CA, USA) following the manufacturer's recommendations.

### 2.10. Immunofluorescence Cell Staining

For immunofluorescence staining, C2C12 cells grown in 24-well plates were washed with PBS and fixed in 4% paraformaldehyde for 10 minutes at 4°C. The fixed cells were permeabilized with 0.25% Triton X-100 for 10 minutes and then blocked with 10% donkey serum in PBST for 1 hour at room temperature. The cells were subsequently incubated with primary antibodies in 2.5% BSA overnight at 4°C. The cells were incubated with Alexa Fluor 488-conjugated donkey anti-mouse and Alexa Fluor 594-conjugated donkey anti-rabbit antibodies for 1 hour. After the cells were stained with DAPI for 5 minutes, micrographs of the cells were taken with a Leica DMi8 microscope and analyzed by ImageJ.

### 2.11. Hoechst 33342 and Propidium Iodide (PI) Double Staining

Cell death was identified by Hoechst 33342/PI double staining. The final concentrations of Hoechst 33342 (Sigma-Aldrich, MO, USA)) and PI (Beyotime, Shanghai, China) in DMEM were 2 *μ*g/ml and 0.5 *μ*g/ml, respectively. Cells seeded in 6-well plates were washed once with PBS gently and then incubated with mixed staining solution at 37°C for 15 minutes, protected from light. After the cells were washed with PBS twice, photographs of the cells were taken immediately with a fluorescence microscope.

### 2.12. Flow Cytometry Analysis

The change in cell size was evaluated using a NovoCyte flow cytometer (ACEA Biosciences, Hangzhou, China). Forward scatter (FSC) is proportional to the size of the cell. Larger cells have stronger FSC signals. Side scatter (SSC) is related to cell granularity and structural complexity. Cells were treated with CoCl_2_ or 2 mM NAC for 24 hours and then digested and centrifuged for 3 minutes at 1000 rpm. After resuspension in DMEM, 3 × 10^4^ cells per sample were tested, and cell debris was excluded by gating. The values of forward scatter height (FSC-H) and side scatter height (SSC-H) were measured and analyzed with NovoExpress 1.2.5 software (ACEA Biosciences, Hangzhou, China).

A cellular ROS kit was used to evaluate ROS levels induced by hypoxia. Briefly, cells were handled following the manufacturer's instructions. The green fluorescence of DCF was detected using the FITC channel at 488 nm excitation and 530 nm emission. For the apoptosis assay, a FITC Annexin V Apoptosis Kit (BD, NJ, USA) was used. Briefly, the cells were handled following the manufacturer's instructions. Green (Annexin) and red (PI) fluorescence was detected using the FITC and PE channels, respectively. The data were analyzed with NovoExpress 1.2.5 software.

### 2.13. siRNA transfection

siRNA targeting mouse GSDMD and the negative control were labeled with CY3 and synthesized by GenePharma (Shanghai, China). The GSDMD siRNA sequences were 5′-GGAUUGAUGAGGAGGAAUUTT -3′ (sense) and 5′-AAUUCCUCCUCAUCAAUCCTT -3′ (antisense). For siRNA transfection, C2C12 cells were seeded in 6-well plates. Lipofectamine RNAiMAX reagent and Opti-MEM (Invitrogen, CA, USA) were used. The final concentration of siRNA was 25 pmol per well. Sixteen hours after transfection, the medium was replaced with a fresh complete medium. Then, CoCl_2_ was added after 24 hours of incubation.

### 2.14. Statistical Analysis

The data in this study were analyzed with GraphPad Prism 5 and presented as the mean ± SD. Comparisons between groups were made using Student's *t*-test or one-way ANOVA with Bonferroni's post hoc test. Statistical differences were considered significant when ^∗^*p* < 0.05, ^∗∗^*p* < 0.01, and ^∗∗∗^*p* < 0.001.

## 3. Results

### 3.1. Increased Pyroptotic Cell Death of Skeletal Muscle in OSA Mice

**Figure 1:**
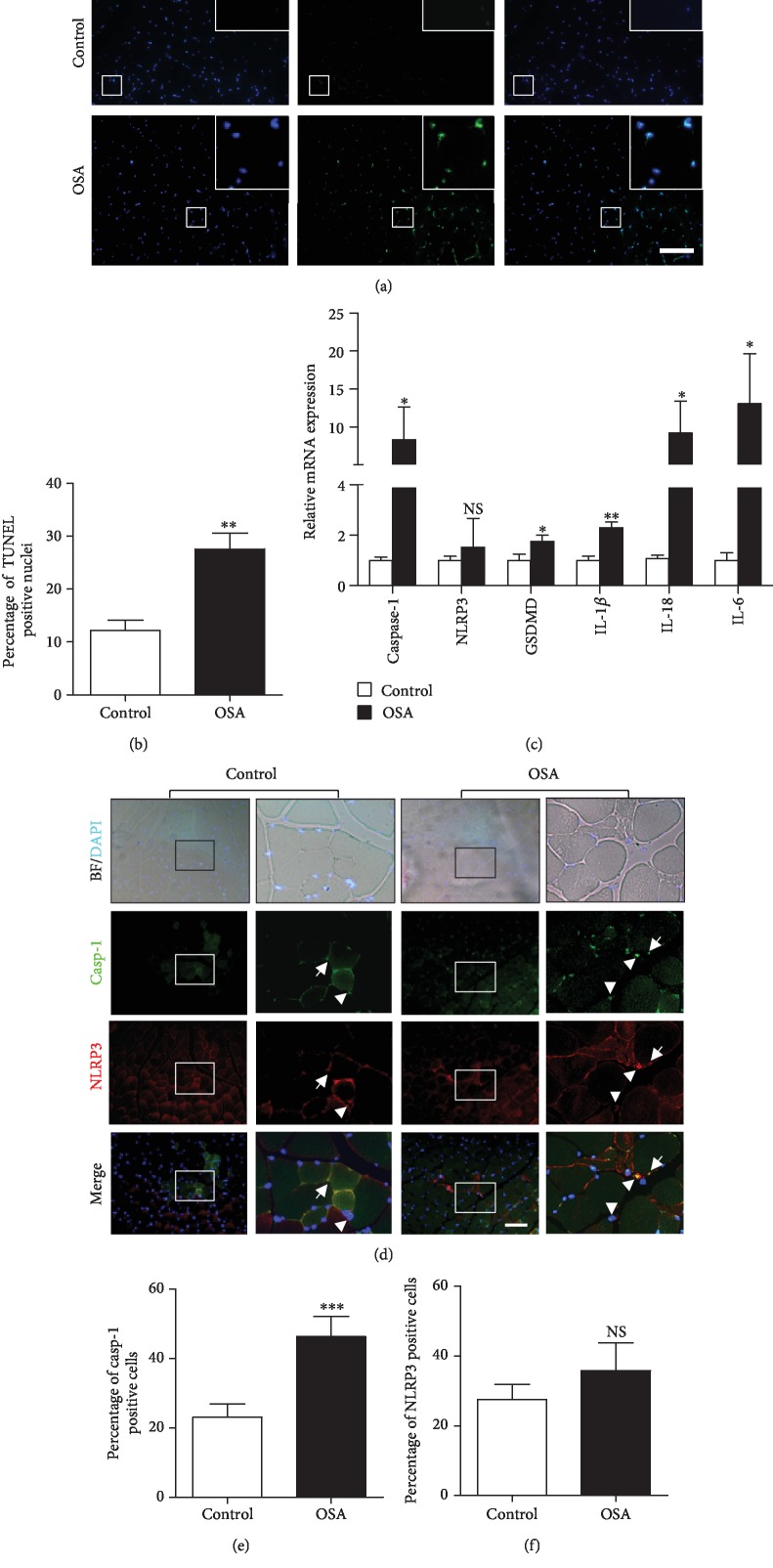
Increase in pyroptotic cell death in the gastrocnemius muscle of OSA mice. (a) TUNEL staining and (b) the percentage of TUNEL-positive nuclei in the gastrocnemius muscles of control and OSA mice. Scale bars = 100 *μ*m. (c) Real-time PCR analysis of relative inflammatory gene expression in the gastrocnemius muscle of OSA mice (*n* = 3). (d–f) Caspase-1 (green) and NLRP3 (red) double staining in control and OSA mouse gastrocnemius sections. The staining results showed more nuclear colocalization of caspase-1 and NLRP3 (white arrow) in OSA mice. The white triangle indicates cytoplasmic colocalization. Scale bars = 100 *μ*m. The data are shown as the mean ± SD. ^∗^*p* < 0.05, ^∗∗^*p* < 0.01, ^∗∗∗^*p* < 0.001; NS: no significant difference.

The OSA mouse model was created as described previously [25]. Mice were treated with chronic intermittent hypoxia for 5 weeks (OSA). To study the effect of hypoxia on cell death in OSA mice, TUNEL staining was performed on gastrocnemius slides. During pyroptosis, the genomic DNA of cells is fragmented and then can be detected by TUNEL staining [26, 27]. The results showed increased TUNEL-positive cells in OSA mice compared with control mice (Figures 1(a) and 1(b)).

Cell pyroptosis is an inflammatory response that is a type of regulated cell death. We then investigated the putative role of hypoxia in pyroptotic cell death in OSA mice. Gastrocnemius muscles were collected, and the expression of pyroptosis-related genes was tested by real-time PCR. Caspase-1 mRNA expression increased by 8.3-fold in OSA mice compared with control mice (Figure 1(c)). GSDMD mRNA expression in OSA mice increased by 1.8-fold when compared with that in control mice. The levels of the inflammatory factors IL-6, IL-1*β*, and IL-18 of OSA were 13-fold, 2.3-fold, and 9.2-fold higher, respectively, than those in control mice (Figure 1(c)). However, the differences in NLRP3 mRNA expression between the two groups were not significant.

We next examined the protein levels of caspase-1 and NLRP3 in the gastrocnemius muscle. Immunofluorescence staining showed a larger caspase-1-positive cell area in OSA mice compared with control mice, while no significant difference in NLRP3-positive cell area was found between the groups (Figures 1(d)–1(f)). Moreover, we found increased nuclear colocalization of caspase-1 and NLRP3 in OSA mice compared with control mice.

### 3.2. CoCl_2_, Which Mimics Hypoxia, Induces Myoblast Cell Swelling, Discoloration, and Inflammatory Responses

Cobalt chloride- (CoCl_2_-) induced hypoxia is one of the most commonly used models in hypoxia research [28]. We used cobalt chloride to mimic hypoxia in vitro. To investigate the effect of hypoxia on C2C12 myoblasts, cells were treated with different doses of CoCl_2_ for 24 and 48 hours. At 400 and 500 *μ*M, a large portion of the cells became round and were floating in the medium. At 100 and 200 *μ*M, the cell morphology was changed, and the cell number was decreased to approximately 50% that of untreated cells. There were no significant differences between the groups at 200 and 300 *μ*M and 400 and 500 *μ*M (Supplementary Figures [Supplementary-material supplementary-material-1] and [Supplementary-material supplementary-material-1]). Thus, we used 200 and 400 *μ*M in the subsequent experiments.

**Figure 2:**
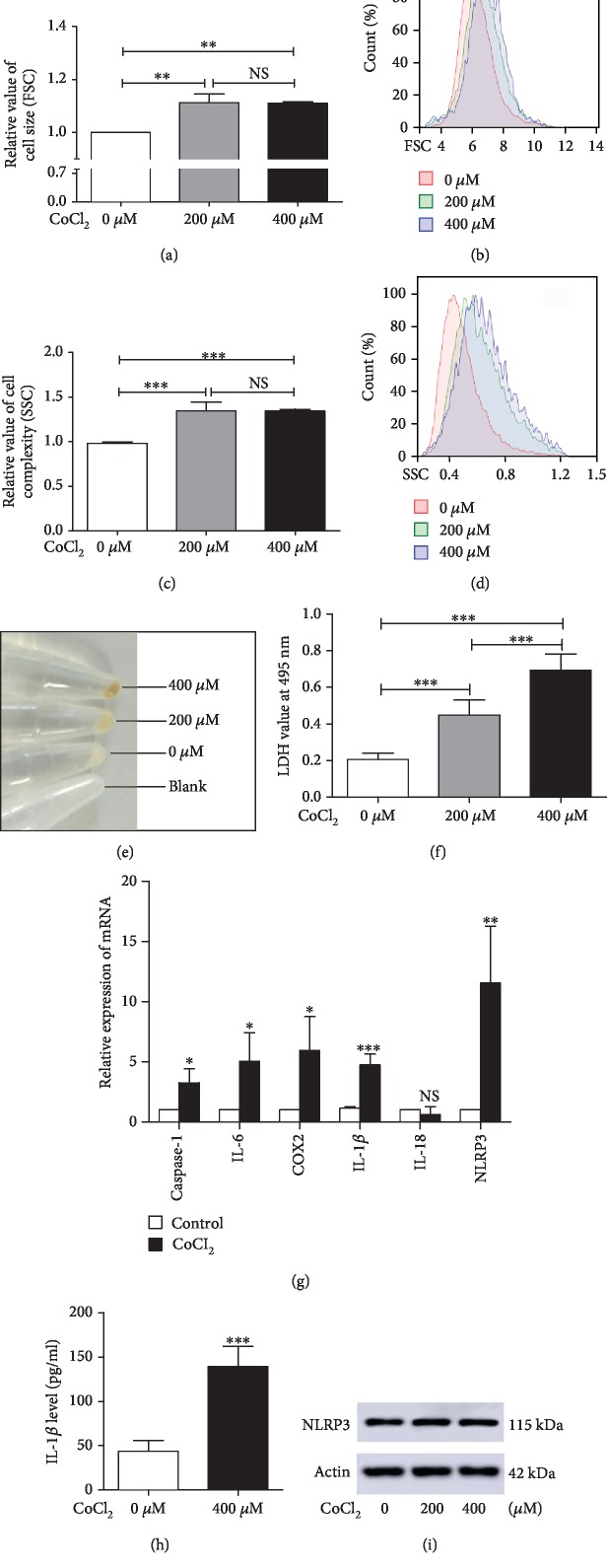
Hypoxia induces the pyroptotic cell death of C2C12 myoblasts. (a–d) Flow cytometry analysis of the relative cell size (FSC) and cell complexity (SSC) of C2C12 cells treated with or without CoCl_2_ for 24 hours. (e) Pellets of C2C12 cells after incubation with or without CoCl_2_ for 24 hours. (f) LDH release was elevated in CoCl_2_-induced hypoxic cells (*n* = 3). (g) Real-time PCR analysis of relative inflammatory gene expression in C2C12 myoblasts (*n* = 3‐5). (h) IL-1*β* level in the supernatant of C2C12 cells treated with or without CoCl_2_ for 24 hours. (i) Western blot analysis of NLRP3 in C2C12 cells 24 hours after CoCl_2_ treatment. The data are shown as the mean ± SD. ^∗^*p* < 0.05, ^∗∗^*p* < 0.01, ^∗∗∗^*p* < 0.001; NS: no significant difference.

Then, we evaluated the putative role of hypoxia in cell physiology by comparing cell size and state using flow cytometry. Compared to control cells, cells that were treated with CoCl_2_ exhibited a larger volume and more complexity (Figures 2(a)–2(d)). In addition, the colors of the cell pellets changed to yellow in the CoCl_2_-treated groups (Figure 2(e)).

To further characterize the effect of hypoxia on cell death, we conducted a LDH assay. The results indicated that C2C12 cells incubated with CoCl_2_ for 24 hours exhibited higher death than that exhibited in normoxic controls (Figure 2(f)). A previous study showed that hypoxia evokes cell apoptosis [9]. We then examined whether apoptotic cell death is involved in this CoCl_2_-induced cell damage. The flow cytometry results showed that increased PI (+)/Annexin V (-) and Annexin V (+) cells in the CoCl_2_-treated groups (Supplementary [Supplementary-material supplementary-material-1]). Meanwhile, the mRNA expression of Bcl-2, an inhibitor of apoptosis, was significantly downregulated in CoCl_2_-treated cells (Supplementary [Supplementary-material supplementary-material-1]). In addition, the apoptosis target lamin B was reduced after CoCl_2_ treatment when compared with that in normoxic controls (Supplementary [Supplementary-material supplementary-material-1]). These results suggested that apoptotic cell death may be partly involved in CoCl_2_-induced cell death.

To further determine whether inflammatory death is involved, inflammatory gene expression was tested. We found that CoCl_2_ treatment, compared with the control, significantly increased the mRNA expression of caspase-1, IL-6, COX2, IL-1*β*, and NLRP3 (Figure 2(g)), while it had no effect on NLRP1, NLRC4, and IL-18 expression (Figure 2(g) and Supplementary [Supplementary-material supplementary-material-1]). More importantly, ELISA results showed that CoCl_2_ treatment increased the IL-1*β* level in the supernatants of C2C12 cells (Figure 2(h)). However, the NLRP3 protein levels were not affected by CoCl_2_ treatment (Figure 2(i) and Supplementary [Supplementary-material supplementary-material-1]). In addition, CoCl_2_ treatment inhibited C2C12 differentiation by downregulating the expression of Pax7, MyoD, and myogenin (Supplementary [Supplementary-material supplementary-material-1]–[Supplementary-material supplementary-material-1]).

### 3.3. Caspase-1 Is Activated in Hypoxia-Induced Pyroptotic Cell Death

**Figure 3:**
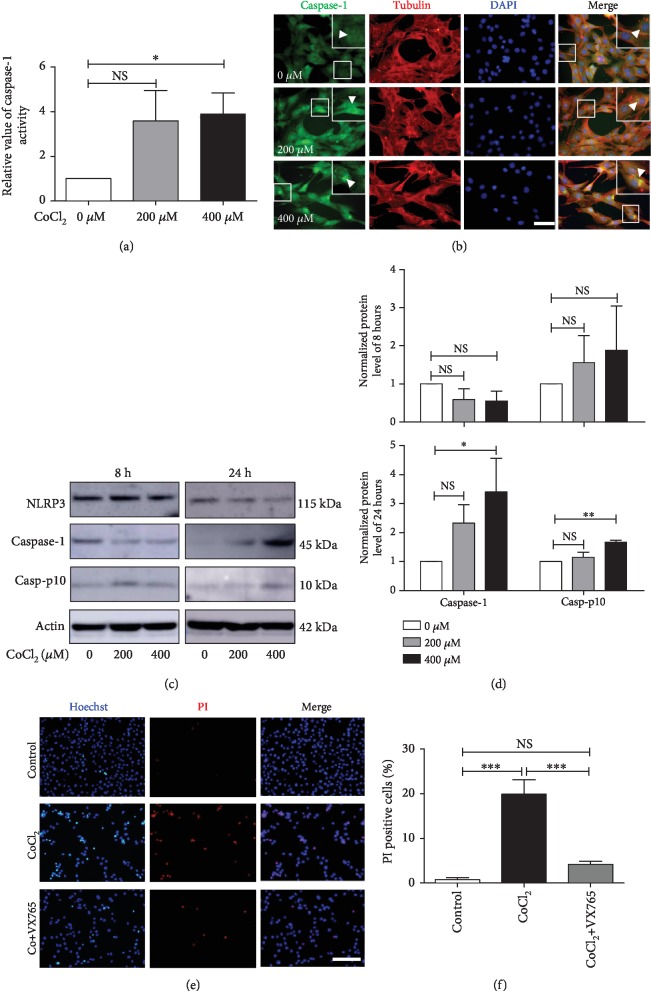
Hypoxia activates caspase-1 in C2C12 cells. (a) Assay of caspase-1 activity in C2C12 cells with or without CoCl_2_ treatment (*n* = 3). (b) Immunofluorescence showed that caspase-1 clustered (arrow) at the nuclear membrane in CoCl_2_-treated cells, but not in control cells. Scale bars = 50 *μ*m. (c, d) Western blot analysis of caspase-1 and cleaved caspase-1 p10 in C2C12 cells after CoCl_2_ treatment for 8 and 24 hours (*n* = 3). (e, f) Hoechst/PI double staining of C2C12 cells. A caspase-1 inhibitor (VX765) ameliorated CoCl_2_-induced cell death. Scale bars = 50 *μ*m. The data are shown as the mean ± SD. ^∗^*p* < 0.05, ^∗∗^*p* < 0.01, ^∗∗∗^*p* < 0.001; NS: no significant difference.

We further examined whether pyroptosis is involved in hypoxia-induced cell death. To that end, we tested caspase-1 activity and expression. Following the treatment of C2C12 cells with CoCl_2_, the caspase-1 activity was 4-fold higher than that in control cells (Figure 3(a)). To explore the functionality of hypoxia-induced caspase-1, we detected its subcellular location in C2C12 cells by immunofluorescence. We found that caspase-1 preferentially existed in the cytoplasm of control cells, whereas CoCl_2_ treatment induced caspase-1 clustering in close proximity to the nuclear membrane after 24 hours of incubation (Figure 3(b)). These results suggested that caspase-1 might play a role in pyroptotic damage to the nuclear membrane.

As active caspase-1 undergoes cleavage, we performed Western blotting to determine the level of cleaved caspase-1. After 8 hours of incubation, the CoCl_2_ groups exhibited lower levels of caspase-1 (45 kDa) and higher levels of cleaved caspase-1 (10 kDa) than those in the control groups, but the differences were not statistically significant (Figures 3(c) and 3(d)). However, CoCl_2_ treatment, especially 400 *μ*M CoCl_2_, increased the protein expression of caspase-1 and cleaved caspase-1 in C2C12 cells after incubation for 24 hours (Figures 3(c) and 3(d)).

To further characterize the requirement of caspase-1 in hypoxia-induced death, C2C12 cells were pretreated with or without a caspase-1 inhibitor (VX765). The Hoechst/PI double staining results indicated that VX765 treatment ameliorated CoCl_2_-induced C2C12 cell death (Figures 3(e) and 3(f)).

### 3.4. GSDMD Is Involved in Hypoxia-Induced Myoblast Pyroptosis

The 53-kDa protein GSDMD is a downstream effector of pyroptosis. It is composed of a functional N-terminal domain (30 kDa) and a self-inhibitory C-terminal domain (23 kDa). GSDMD is cleaved into GSDMD-p30, which forms pores in the cytoplasm membrane during pyroptosis [16]. Therefore, we evaluated its involvement in hypoxia-induced cell death.

**Figure 4:**
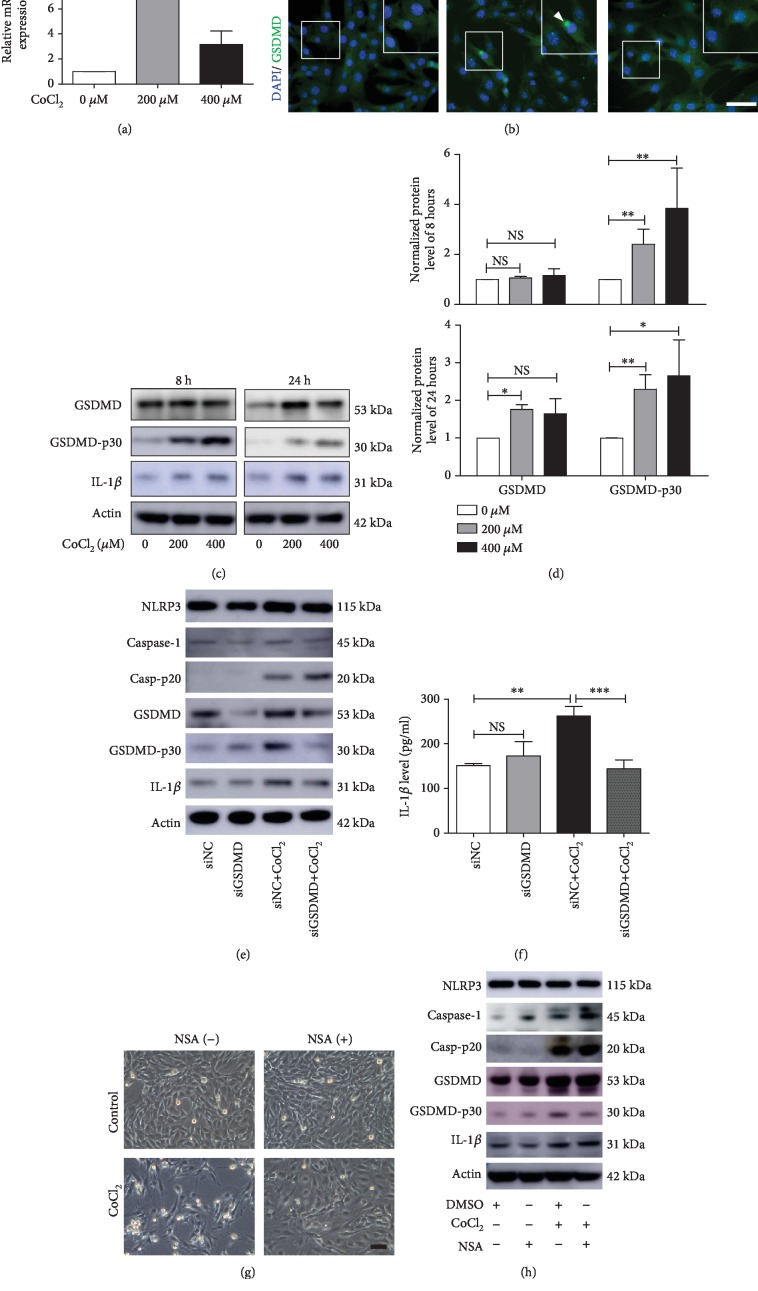
GSDMD is involved in hypoxia-induced myoblast pyroptosis. (a) Real-time PCR analysis of relative GSDMD mRNA expression in C2C12 myoblasts with or without hypoxia (CoCl_2_) treatment for 24 hours (*n* = 3). (b) Immunofluorescence staining for GSDMD in C2C12 myoblasts with or without CoCl_2_ treatment for 24 hours. GSDMD clustered (arrow) at the nuclear membrane in hypoxic cells in the 200 *μ*M CoCl_2_ group. Scale bars = 50 *μ*m. (c, d) Western blot analysis of GSDMD and GSDMD-p30 in C2C12 cells with or without CoCl_2_ treatment for 8 and 24 hours. (e) After transfection with siGSDMD, pyroptosis-related protein expression in C2C12 cells treated with or without CoCl_2_ for 24 hours. NC indicates negative control. (f) IL-1*β* level in the supernatants of the transfected cells treated with or without CoCl_2_ for 24 hours. NC indicates negative control. (g) Effects of NSA (1 *μ*M) treatment followed by CoCl_2_ treatment in C2C12 cells for 24 hours. (h) Western blot analysis of pyroptosis-related protein expression after NSA treatment. The data are shown as the mean ± SD. ^∗^*p* < 0.05, ^∗∗^*p* < 0.01, ^∗∗∗^*p* < 0.001; NS: no significant difference.

Real-time PCR showed that 200 *μ*M CoCl_2_ for 24 hours significantly increased GSDMD mRNA expression in C2C12 cells. Treatment with 400 *μ*M CoCl_2_ induced higher expression than that in control cells, although the differences were not statistically significant (Figure 4(a)). As the location of caspase-1 was changed in hypoxic cells, we next tested the subcellular distribution of GSDMD by immunofluorescence staining as well. We found that GSDMD was clustered in close proximity to the nuclear membrane in the 200 *μ*M CoCl_2_-treated groups (Figure 4(b)).

Then, we determined the changes in GSDMD protein levels using Western blotting. Eight hours after CoCl_2_ treatment, the levels of GSDMD-p30 were significantly increased compared with those in the control group, while there was no significant change in GSDMD levels in the CoCl_2_-treated groups. Twenty-four hours after CoCl_2_ treatment, the levels of both GSDMD and GSDMD-p30 were upregulated (Figures 4(c) and 4(d)). To further investigate the function of GSDMD, we knocked down GSDMD with siRNA in C2C12 cells (Supplementary Figures [Supplementary-material supplementary-material-1] and [Supplementary-material supplementary-material-1]). We found that knockdown of GSDMD decreased the level of released IL-1*β* induced by CoCl_2_ treatment (Figures 4(e) and 4(f)). The results suggested that GSDMD is involved in CoCl_2_-induced IL-1*β* release.

To further explore GSDMD activation during C2C12 pyroptosis, a GSDMD inhibitor, necrosulfonamide (NSA), was used. NSA can directly bind to GSDMD and inhibit pore formation by GSDMD-p30 [29]. To investigate the role of NSA in CoCl_2_-induced pyroptosis, we pretreated C2C12 cells with 1 *μ*M NSA for 2 hours and then with CoCl_2_ for 24 hours. The results showed that NSA significantly protected the cells from CoCl_2_-induced death (Figure 4(g) and Supplementary Figures [Supplementary-material supplementary-material-1] and [Supplementary-material supplementary-material-1]). Recently, NSA was found to inhibit caspase-1 activation [30], which is the upstream of GSDMD. In these experiments, we found that NSA reduced the cleaved caspase-1 levels in the normoxic groups but had no such effect in CoCl_2_-induced cells (Figure 4(h)).

### 3.5. ROS May Affect Hypoxia-Induced Pyroptosis in Myoblasts

**Figure 5:**
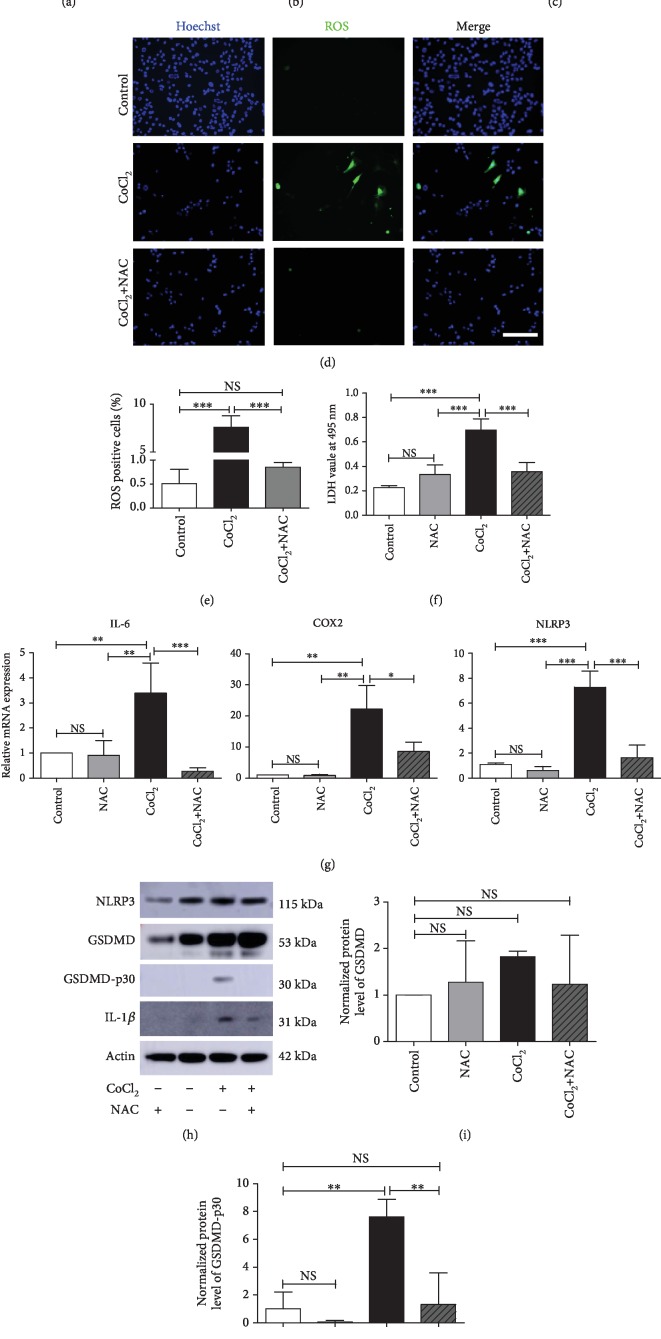
ROS mediate hypoxia-induced pyroptosis in myoblasts. (a) Pellets of C2C12 cells after treatment with or without 2 mM NAC under normoxic control and CoCl_2_-induced hypoxic conditions for 24 hours. (b, c) Flow cytometry analysis of the relative cell size (FSC) and cell complexity (SSC) of C2C12 cells treated with or without NAC under normoxic and CoCl_2_-induced hypoxic conditions for 24 hours (*n* = 3). (d, e) ROS assay in C2C12 cells treated with or without NAC under normoxic and CoCl_2_-induced hypoxic conditions for 24 hours. Scale bars = 50 *μ*m. (f) NAC treatment decreased the level of LDH release induced by CoCl_2_ in C2C12 cells (*n* = 3). (g) Real-time PCR analysis of relative inflammatory gene expression in C2C12 myoblasts treated with or without NAC for 8 hours (*n* = 3). (h–j) Western blot analysis of pyroptosis-related protein expression in C2C12 cells treated with or without NAC under normoxic control and CoCl_2_-induced hypoxic conditions for 24 hours. The data are shown as the mean ± SD. ^∗^*p* < 0.05, ^∗∗^*p* < 0.01, ^∗∗∗^*p* < 0.001; NS: no significant difference.

Excessive ROS can cause damage to tissues and cells. Our previous study showed that an increased level of ROS impairs the fatigue resistance of the genioglossus [6]. To investigate the role of ROS in hypoxia-induced myoblast pyroptosis, we pretreated C2C12 cells with different doses of NAC for 2 hours and then with CoCl_2_ for 24 hours. The results showed that 2 mM NAC significantly protected the cells from CoCl_2_-induced death (Supplementary [Supplementary-material supplementary-material-1]). Meanwhile, NAC inhibited cell yellowing caused by CoCl_2_ treatment (Figure 5(a)). Then, we investigated the role of ROS in hypoxia-induced cell morphology. C2C12 cells were analyzed by flow cytometry. CoCl_2_ treatment increased the cell size (FSC) and cell complexity (SSC) of C2C12 cells by 15% and 50%, respectively. NAC treatment significantly ameliorated cell swelling and lowered the complexity induced by CoCl_2_ but had no effect under normoxic conditions (Figures 5(b) and 5(c) and Supplementary [Supplementary-material supplementary-material-1]).

CoCl_2_ treatment increased cellular ROS levels, which were significantly lowered by NAC (Figures 5(d) and 5(e) and Supplementary [Supplementary-material supplementary-material-1]). Following NAC treatment, LDH release decreased to 51% of that in cells treated with CoCl_2_ (Figure 5(f)). We then examined the effect of NAC on inflammatory gene expression. Real-time PCR results indicated that NAC significantly downregulated the mRNA levels of IL-6, COX2, NLRP3, and IL-1*β* in cells treated with CoCl_2_ (Figure 5(g) and Supplementary [Supplementary-material supplementary-material-1]). To evaluate whether ROS were involved in hypoxia-induced pyroptosis, C2C12 cells were treated with or without NAC. Western blot analyses showed that the expression level of GSDMD-p30 was significantly reduced by NAC compared with that in cells treated with CoCl_2_. However, GSDMD itself was not downregulated by NAC (Figures 5(h)–5(j)). In addition, NAC did not regulate NLRP3 expression in either the control or CoCl_2_-treated groups (Figure 5(h) and Supplementary [Supplementary-material supplementary-material-1]).

### 3.6. Hypoxia Activates the NF-*κ*B Signaling Pathway in Myoblasts

**Figure 6:**
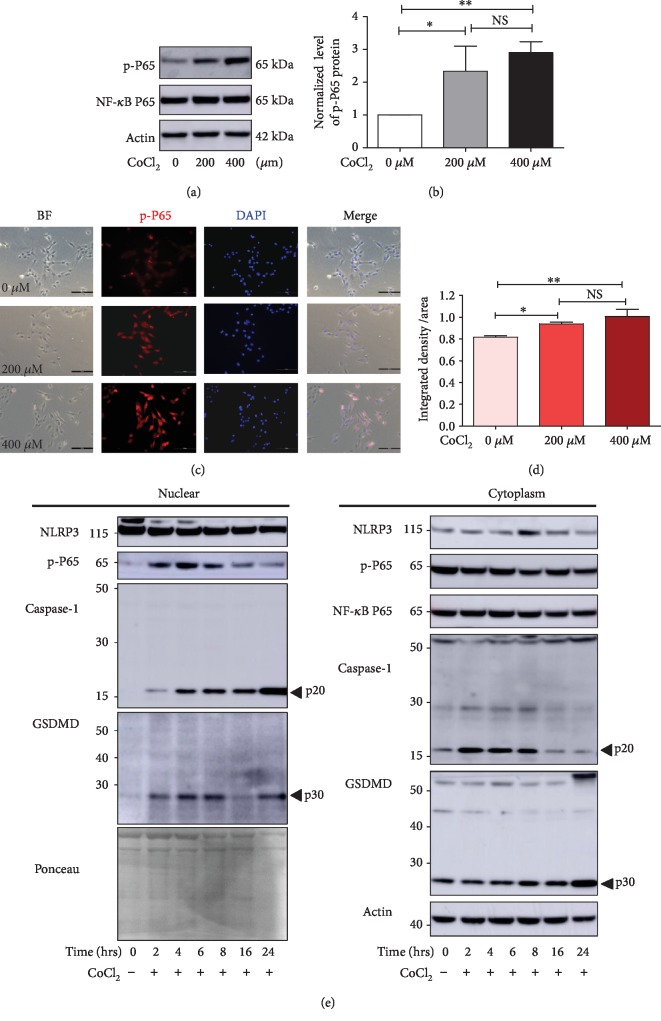
Hypoxia activates the NF-*κ*B P65 signaling pathway. (a, b) Western blot analysis of NF-*κ*B P65 phosphorylation in C2C12 cells after CoCl_2_ treatment for 8 hours (*n* = 3). (c, d) Immunofluorescence in C2C12 cells showing p-P65 upregulation with or without 2 hours of CoCl_2_ treatment. Scale bars = 100 *μ*m. (e) Western blot analysis of nuclear and cytoplasmic protein expression of pyroptosis proteins at different time points after CoCl_2_ treatment. The data are shown as the mean ± SD. ^∗^*p* < 0.05, ^∗∗^*p* < 0.01; NS: no significant difference.

The NF-*κ*B signaling pathway is closely related to oxidative stress and inflammatory diseases. To investigate whether NF-*κ*B is involved in hypoxia-induced cell pyroptosis, C2C12 cells were incubated with or without CoCl_2,_ and NF-*κ*B P65 phosphorylation was detected by Western blotting. The p-P65 level was significantly upregulated 8 hours after CoCl_2_ treatment (Figures 6(a) and 6(b)), but no changes were observed within 60 minutes (Supplementary [Supplementary-material supplementary-material-1]). To further characterize the subcellular localization of hypoxia-induced P65 phosphorylation, we detected p-P65 distribution in C2C12 cells by immunofluorescence. We found that p-P65 was diffused in both the cytoplasm and nuclei of control cells, whereas, 2 hours after CoCl_2_ treatment, p-P65 expression was increased (Figures 6(c) and 6(d)). Furthermore, we evaluated p-P65 levels both in the nucleus and cytoplasm at different time points after CoCl_2_ treatment. Nuclear translocation was found to have peaked at 4 hours (Figure 6(e)).

We then compared differences in the expression of caspase-1. Surprisingly, cleaved caspase-1 (casp-p20) and GSDMD-p30 were found in both the nucleus and cytoplasm 2 hours after CoCl_2_ treatment, while caspase-1 and GSDMD were only detected in the cytoplasm of C2C12 cells (Figure 6(e)). Casp-p20 expression in the nuclei was upregulated gradually from 2 to 24 hours after CoCl_2_ treatment. In the cytoplasm, higher levels of casp-p20 were detected from 2 to 8 hours after CoCl_2_ treatment (Figure 6(e)).

### 3.7. NAC May Protect C2C12 Cells from Hypoxia-Induced Damage through the ROS/NF-*κ*B/HIF-1*α* Pathway

**Figure 7:**
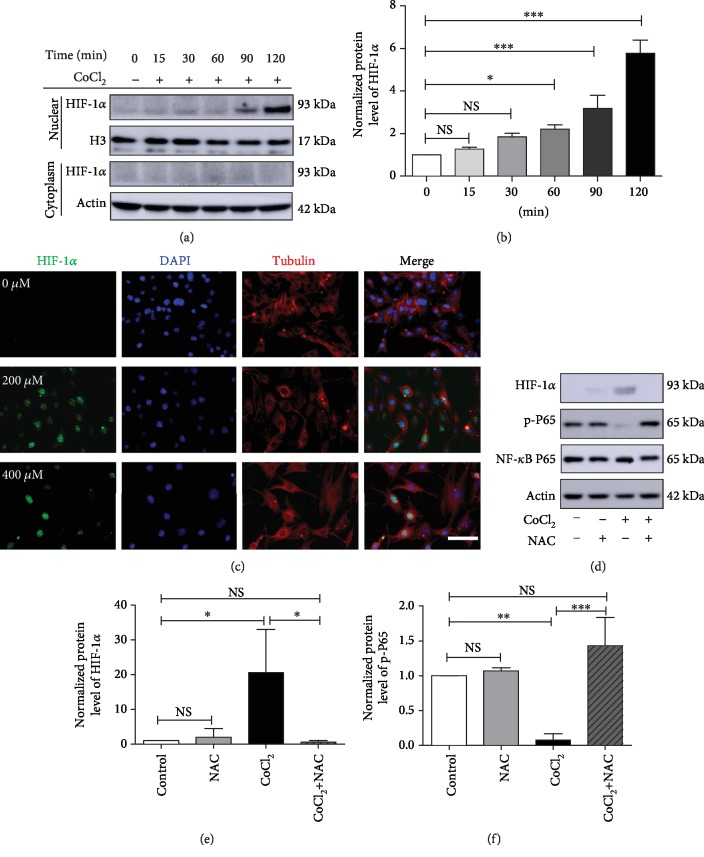
NAC downregulates HIF-1*α* expression activated by CoCl_2_ in myoblasts. (a, b) Western blot analysis of HIF-1*α* in C2C12 cells at different time points after CoCl_2_ treatment. (c) Immunofluorescence staining for HIF-1*α* in C2C12 myoblasts treated with or without CoCl_2_ for 24 hours. CoCl_2_ treatment induced HIF-1*α* nuclear expression. Scale bars = 50 *μ*m. (d–f) Western blot analysis of HIF-1*α* and phosphorylated NF-*κ*B P65 in C2C12 cells after CoCl_2_ treatment for 24 hours (*n* = 3). The data are shown as the mean ± SD. ^∗^*p* < 0.05, ^∗∗^*p* < 0.01, ^∗∗∗^*p* < 0.001; NS: no significant difference.

We previously found that HIF-1*α* plays an important role in OSA [8]. To investigate crosstalk between HIF-1*α* and NF-*κ*B in hypoxia-induced C2C12 cells, we first detected HIF-1*α* expression following 400 *μ*M CoCl_2_ treatment. The level of HIF-1*α* in the nucleus increased gradually 2 hours after CoCl_2_ treatment, while no bands were detected in the cytoplasm of C2C12 cells (Figures 7(a) and 7(b)). Then, we performed immunofluorescence staining for HIF-1*α*. The results confirmed its nuclear localization in the CoCl_2_-treated groups, while no fluorescence was detected in control cells (Figure 7(c)).

To further elucidate the role of ROS in HIF-1*α* activation, we then treated C2C12 cells with or without NAC under both normoxic and hypoxic conditions. Western blot analyses showed that NAC decreased the protein level of HIF-1*α* induced by CoCl_2_ treatment (Figures 7(d) and 7(e)). Moreover, NAC rescued p-P65 expression 24 hours after CoCl_2_ treatment (Figures 7(d) and 7(f)). HIF-1*α* is ubiquitously expressed in mammalian cells and is rapidly degraded by the intracellular ubiquitin-proteasome pathway under normoxic conditions. We further explored the effect of NAC on the mRNA expression of HIF-1*α*. The data showed that CoCl_2_ treatment reduced the mRNA expression of HIF-1*α*. However, there was no significant difference in the HIF-1*α* expression after NAC treatment in either normoxic or CoCl_2_-treated cells (Supplementary [Supplementary-material supplementary-material-1]).

## 4. Discussion

This study showed that hypoxia induced excessive ROS formation and inflammatory caspase-1 activation, leading to the pyroptotic cell death of myoblasts in OSA. In vivo, more TUNEL-positive cells were observed in the skeletal muscle of OSA mice. Our IHC and real-time PCR studies revealed more caspase-1 expression in the muscles than in the muscles of control mice. Moreover, the expression of pyroptosis-related gene expressions, including GSDMD, IL-1*β*, and IL-18, was also upregulated in the muscles of OSA mice. Myoblasts are activated muscle satellite cells residing in a hypoxic niche. These muscle progenitor cells are responsible for skeletal myogenesis and regeneration. Although moderate hypoxia promotes muscle myogenesis [31], hypoxia leads to muscle injury in patients with hypoxia-related diseases such as OSA [32, 33]. More importantly, OSA has been reported to be an independent risk factor for cardiovascular diseases and metabolic disorders [3]. Therefore, hypoxia-induced cell death may play a crucial role in OSA and its comorbidities.

Cell pyroptosis, unlike apoptosis and autophagy, is an inflammatory form of cell death induced by the inflammasome. Inflammasomes such as the NLRP3 inflammasome sense extracellular or intracellular stimuli and activate caspase-1, leading to the formation of functional GSDMD and the release of the proinflammatory cytokines IL-1*β* and IL-18 [11]. In our in vitro study, we found that hypoxia activated caspase-1 and GSDMD and ultimately contributed to cell pyroptosis in myoblasts. Previous studies have shown that hypoxia induces apoptosis and autophagy in myoblasts [9, 13]. We also found that, after CoCl_2_-mediated hypoxia, some cells were apoptotic. Interestingly, a recent study reported that apoptosis signaling induces a parallel pathway to trigger pyroptosis in macrophages [34]. Moreover, our data showed that hypoxia increased the mRNA expression of several inflammation-related genes, including IL-6, NLRP3, COX2, and IL-1*β*. Thus, hypoxia-induced pyroptosis may be related to systemic inflammation, as in OSA patients, or it may be a part of the cellular mechanisms of the pathophysiology of OSA.

Inflammasomes are multiprotein complexes that can induce pyroptotic cell death. Of the members of the nucleotide-binding domain and leucine-rich repeat receptor (NLR) family, NLRP3 is the best characterized. Previous studies have shown that hypoxia could regulate NLRP3 expression [35, 36]. Our results showed that hypoxia upregulated the mRNA expression of NLRP3 remarkably but not the NLRP1 or NLRC4 genes in myoblasts. However, no increase in NLRP3 expression was found in either the muscles in OSA or in hypoxic cells at the protein level, suggesting possible negative posttranscriptional modulation in C2C12 cells. However, the serum levels of the NLRP3 inflammasome are not different between OSA patients and healthy controls [37]. The kidneys of NLRP3^−/−^ mice are protected from OSA-induced injury and oxidative stress [38]. Interestingly, we detected more NLRP3 and caspase-1 nuclear colocalization in OSA skeletal muscle. Western blotting of C2C12 cells further confirmed NLRP3 and cleaved caspase-1 nuclear expression. These results are in line with a study showing that NLRP3 and caspase-1 are located in the cell nucleus [39, 40]. In addition, caspase-1 was observed to be clustered at the nuclear membrane in hypoxic cells and was preferentially distributed in the cytoplasm of normoxic cells. In agreement with our study, caspase-1 was previously observed in a cluster at the nuclear membrane that may have been associated with nuclear lysis [41]. The abnormal distribution of caspase-1 and NLRP3 induced by hypoxia may be partly associated with their regulatory effects on gene expression.

GSDMD is the downstream effector during pyroptosis and is cleaved into GSDMD-p30 to form pores in the cytoplasm. Subsequently, the rupturing of the membrane results in the loss of the physical integrity of the cell, leading to cell swelling. In the present study, we applied flow cytometry to detect cell size and intracellular complexity. The increased complexity in the hypoxia milieu may be associated with organelle damage and concomitant content release during pyroptosis [42]. Importantly, CoCl_2_-induced hypoxia caused C2C12 cell swelling and increased internal complexity, which was rescued by an ROS scavenger (NAC), suggesting that ROS overproduction follows hypoxia in cells. Cell size is related to the homeostatic balance of anabolic and catabolic processes and is adaptive to ambient stimuli [43]. Hypoxia increases the cell volume of glioblastoma multiforme cells by 20% [44]. In addition, C2C12 cells became yellow after culture under hypoxic conditions. Importantly, NAC protected against C2C12 cell yellowing induced by hypoxia. It is unknown why and how hypoxia induces the yellowing of cells. We speculate that intracellular ROS may be involved in the process of color alteration.

Studies have demonstrated increased ROS levels and higher oxidative stress in OSA [5, 6, 37]. Oxidative stress was previously shown to mediate the pyroptosis of cardiomyocytes, macrophages, and neuronal cells [18–20]. We found that hypoxia stimulated ROS generation and accumulation in C2C12 myoblasts, while NAC significantly ameliorated the hypoxia-induced cell death of myoblasts. NAC markedly decreased the levels of inflammatory genes, including IL-1*β*, IL-6, and NLRP3. Moreover, NAC dramatically reduced the levels of cleaved GSDMD without affecting GSDMD expression. Several studies have shown that targeting ROS can attenuate cardiovascular injury in OSA [45–47]. Therefore, antioxidant treatment may be used as an alternative therapeutic strategy to diminish oxidative stress injury and prevent some comorbidities of OSA.

Our signaling studies showed that hypoxia activated the NF-*κ*B and HIF-1*α* pathways. NF-*κ*B and HIF-1*α* are key transcription factors involved in inflammation and hypoxic diseases, respectively. Inflammation mainly activates NF-*κ*B, while hypoxia is closely related to the HIF-1 signaling pathway [24]. Our results showed that NF-*κ*B P65 phosphorylation was upregulated after hypoxia and that HIF-1*α* stabilization and nuclear expression were detected in myoblasts. We also previously showed that the downregulation of HIF-1*α* has protective effects on genioglossus myoblasts and genioglossus fatigue resistance [8]. Additionally, our results showed that NAC inhibited hypoxia-induced HIF-1*α* stabilization and nuclear expression and partially restored NF-*κ*B signaling. It should be noted that there are inconsistent levels of p-P65 expression after CoCl_2_ treatment. We speculate that it may be caused by the different ways of sample preparation. Our data are in agreement with the crosstalk between HIF-1*α* and the NF-*κ*B signaling pathway. These results provide a possible explanation of systemic inflammation in OSA patients.

**Figure 8:**
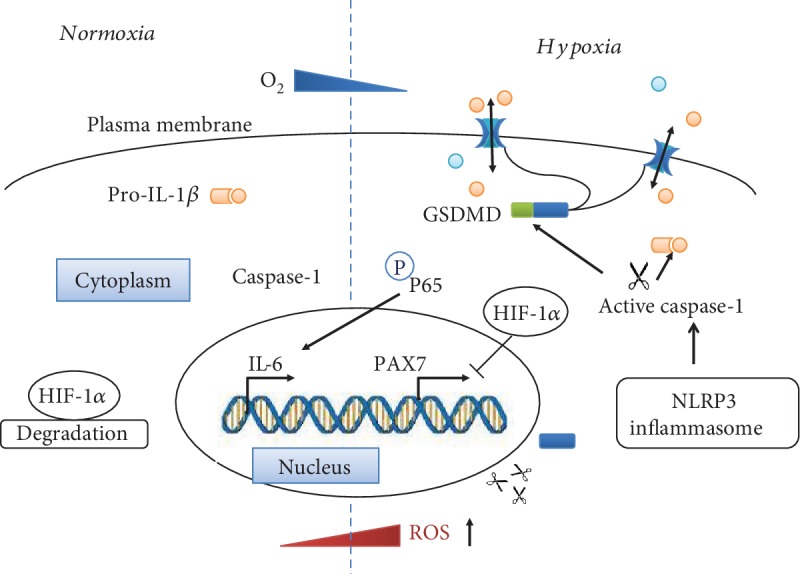
Schematic diagram of hypoxia-induced pyroptosis in myoblasts. Hypoxia increases ROS production, which activates the NLRP3 inflammasome to release activated caspase-1. Activated caspase-1 cleaves GSDMD into GSDMD-p30 and pro-IL-1*β* into mature IL-1*β*, leading to membrane rupture and inflammatory responses. Moreover, hypoxia induces the nuclear translocation of the key transcriptional factor HIF-1*α* and activates the NF-*κ*B signaling pathway, regulating the transcription of myogenic and inflammatory genes.

To summarize our findings, we showed increased pyroptotic markers in muscles in OSA and hypoxia-evoked pyroptotic responses in myoblast cells. However, we cannot discount other types of cell death of myoblasts during the development and maintenance of OSA. Our mechanistic studies suggested that hypoxia-induced myoblast pyroptosis was mediated by ROS overproduction and HIF-1*α* and NF-*κ*B activation (Figure 8).

## 5. Conclusion

In conclusion, our study indicates the involvement of pyroptotic cell death in myoblasts in OSA, and targeting ROS may ameliorate hypoxia-induced cell death and prevent cells from injury due to oxidative stress in OSA.

## Figures and Tables

**Table 1:** Specific primers used for qRT-PCR.

Gene	Forward primer (5′-3′)	Reverse primer (5′-3′)
18S	GTAACCCGTTGAACCCCATT	CCATCCAATCGGTAGTAGCG
NLRP3	ATTACCCGCCCGAGAAAGG	TCGCAGCAAAGATCCACACAG
COX2	TTCAACACACTCTATCACTGGC	AGAAGCGTTTGCGGTACTCAT
IL-6	GAGGATACCACTCCCAACAGACC	AAGTGCATCATCGTTGTTCATACA
Caspase-1	AATACAACCACTCGTACACGTC	AGCTCCAACCCTCGGAGAAA
GSDMD	CCATCGGCCTTTGAGAAAGTG	ACACATGAATAACGGGGTTTCC
IL-1*β*	GCAACTGTTCCTGAACTCAACT	ATCTTTTGGGGTCCGTCAACT
Pax7	TCTCCAAGATTCTGTGCCGAT	CGGGGTTCTCTCTCTTATACTCC
MyoD	CCACTCCGGGACATAGACTTG	AAAAGCGCAGGTCTGGTGAG
Myogenin	GAGACATCCCCCTATTTCTACCA	GCTCAGTCCGCTCATAGCC

## Data Availability

The data used to support the findings of this study are included within the article.

## Abbreviations

OSA: Obstructive sleep apnea
ROS: Reactive oxygen species
GSDMD: Gasdermin D
NLRP: Nod-like receptor protein
HIF-1*α*: Hypoxia-inducible factor-1*α*
FSC: Forward scatter
SSC: Sideward scatter
NSA: Necrosulfonamide
COX2: Cyclooxygenase 2
IL: Interleukin
LDH: Lactate dehydrogenase
NAC: N-Acetyl-L-cysteine
RCD: Regulated cell death
siRNA: Small interfering RNA.
TUNEL: TdT-mediated dUTP nick-end.

## Supplementary Materials

Supplementary Materials Figure S 1: (a, b) C2C12 cells were treated with different doses of CoCl_2_ for 24 and 48 hours. (c) Flow cytometry analysis of PI/Annexin V staining of C2C12 cells treated with or without 400 *μ*M CoCl_2_ for 24 hours. (d) Real-time PCR analysis of relative Bcl-2 and Bax mRNA expression in C2C12 myoblasts treated with or without 400 *μ*M CoCl_2_ for 24 hours. (e) Western blot analysis of the expression of lamin B1 after 400 *μ*M CoCl_2_ treatment. (f) Real-time PCR analysis of relative NLRP1 and NLRC4 mRNA expression in C2C12 myoblasts treated with or without 400 *μ*M CoCl_2_ for 24 hours. (g) Quantification of NLRP3 protein expression (*n* = 3). The data are shown as the mean ± SD. ^∗^*p* < 0.05; ^∗∗∗^*p* < 0.001; NS: no significant difference. Figure S 2: (a) Real-time PCR analysis of relative Pax7, MoyD, and myogenin mRNA expression in C2C12 myoblasts treated with or without 400 *μ*M CoCl_2_ for 24 hours. (b, c) Hypoxia inhibited myotube formation. C2C12 cells underwent myogenic differentiation for 5 days and were then treated with CoCl_2_ for 48 hours. (d, e) C2C12 cells after transfection with siNC or siGSDMD. NC indicates negative control. (f, g) Hoechst/PI double staining of C2C12 cells. A GSDMD inhibitor (NSA) partly inhibited hypoxia-induced C2C12 cell death. Scale bars = 50 *μ*m. Figure S3: (a) Effects of NAC treatment (0-2 mM) followed by CoCl_2_ treatment in C2C12 cells for 24 hours. At 2 mM, NAC remarkably protected against cell death evoked by CoCl2 treatment. (b) Flow cytometry analysis of the relative cell size (FSC) and cell complexity (SSC) of C2C12 cells treated with or without NAC under normoxic control and CoCl_2_-induced hypoxic conditions for 24 hours. (c) Flow cytometry analysis of ROS levels (FITC channel) in C2C12 cells treated with or without NAC under normoxic control and CoCl_2_-induced hypoxic conditions for 24 hours. (d) Gel electrophoresis of IL-1*β* after real-time PCR amplification treated with or without NAC under normoxic control and CoCl_2_-induced hypoxic conditions for 24 hours. (e) Quantitative analysis of NLRP3 protein expression in C2C12 cells treated with or without NAC under normoxic control and CoCl_2_-induced hypoxic conditions for 24 hours. (f) CoCl_2_ treatment did not affect the expression level of phosphorylated NF-*κ*B P65 in C2C12 cells within 60 minutes. (g) Real-time PCR analysis of relative HIF-1*α* mRNA expression upon treatment with or without NAC (*n* = 3). The data are shown as the mean ± SD. ^∗∗^*p* < 0.01; NS: no significant difference.
