# Supplementary material for: Hypoxia-Induced ROS Contribute to Myoblast Pyroptosis during Obstructive Sleep Apnea via the NF-κB/HIF-1α Signaling Pathway
Source: Oxid Med Cell Longev. 2019 Dec 11;2019:4596368. doi: 10.1155/2019/4596368 (PMC6927050; doi:10.1155/2019/4596368)
Supplement: Supplementary Materials — Figure S 1: (a, b) C2C12 cells were treated with different doses of CoCl2 for 24 and 48 hours. (c) Flow cytometry analysis of PI/Annexin V staining of C2C12 cells treated with or without 400 μM CoCl2 for 24 hours. (d) Real-time PCR analysis of relative Bcl-2 and Bax mRNA expression in C2C12 myoblasts treated with or without 400 μM CoCl2 for 24 hours. (e) Western blot analysis of the expression of lamin B1 after 400 μM CoCl2 treatment. (f) Real-time PCR analysis of relative NLRP1 and NLRC4 mRNA expression in C2C12 myoblasts treated with or without 400 μM CoCl2 for 24 hours. (g) Quantification of NLRP3 protein expression (n = 3). The data are shown as the mean ± SD. ∗p < 0.05; ∗∗∗p < 0.001; NS: no significant difference. Figure S 2: (a) Real-time PCR analysis of relative Pax7, MoyD, and myogenin mRNA expression in C2C12 myoblasts treated with or without 400 μM CoCl2 for 24 hours. (b, c) Hypoxia inhibited myotube formation. C2C12 cells underwent myogenic differentiation for 5 days and were then treated with CoCl2 for 48 hours. (d, e) C2C12 cells after transfection with siNC or siGSDMD. NC indicates negative control. (f, g) Hoechst/PI double staining of C2C12 cells. A GSDMD inhibitor (NSA) partly inhibited hypoxia-induced C2C12 cell death. Scale bars = 50 μm. Figure S3: (a) Effects of NAC treatment (0-2 mM) followed by CoCl2 treatment in C2C12 cells for 24 hours. At 2 mM, NAC remarkably protected against cell death evoked by CoCl2 treatment. (b) Flow cytometry analysis of the relative cell size (FSC) and cell complexity (SSC) of C2C12 cells treated with or without NAC under normoxic control and CoCl2-induced hypoxic conditions for 24 hours. (c) Flow cytometry analysis of ROS levels (FITC channel) in C2C12 cells treated with or without NAC under normoxic control and CoCl2-induced hypoxic conditions for 24 hours. (d) Gel electrophoresis of IL-1β after real-time PCR amplification treated with or without NAC under normoxic control and CoCl2-induced hypoxic conditions for 24 [file 4596368.f1.pdf]

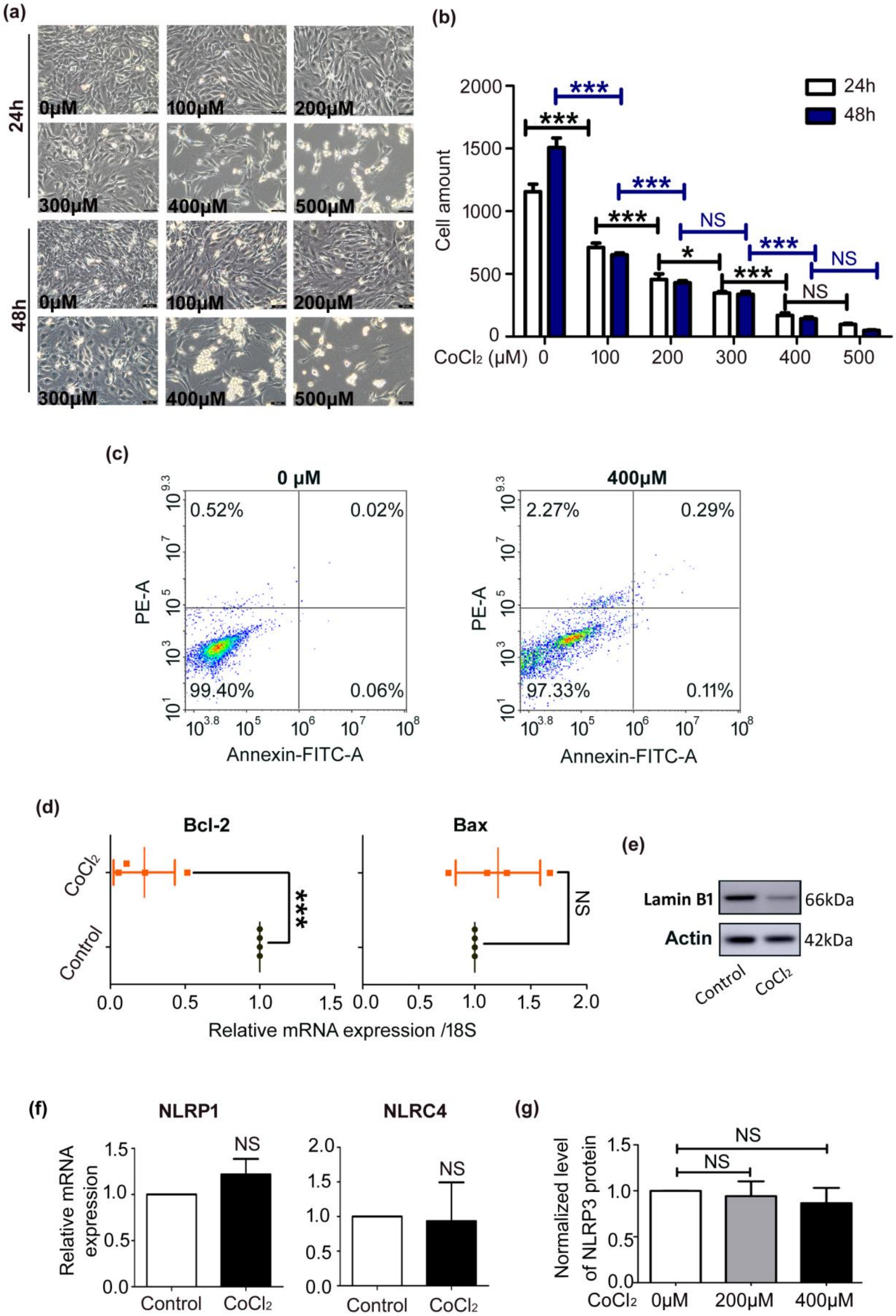

(a)
24h
48h
0μM
100μM
200μM
300μM
400μM
500μM
(b)
24h
48h
Cell amount
2000
1500
1000
500
0
***
*
NS
CoCl2 (μM) 0 100 200 300 400 500
(c)
0 μM
400μM
0.52%
0.02%
99.40%
0.06%
2.27%
0.29%
97.33%
0.11%
PE-A
Annexin-FITC-A
(d)
Bcl-2
Bax
CoCl2
Control
Relative mRNA expression /18S
(e)
Lamin B1
Actin
66kDa
42kDa
Control
CoCl2
(f)
NLRP1
NLRC4
Relative mRNA expression
Control
CoCl2
(g)
Normalized level of NLRP3 protein
CoCl2 0μM 200μM 400μM

FIGURE S 1: (a)-(b) C2C12 cells were treated with different doses of $CoCl_2$ for 24 and 48 hours. (c) Flow cytometry analysis of PI/Annexin V staining of C2C12 cells treated with or without 400 μM $CoCl_2$ for 24 hours. (d) Real-time PCR analysis of relative Bcl-2 and Bax mRNA expression in C2C12 myoblasts treated with or without 400 μM $CoCl_2$ for 24 hours. (e) Western blot analysis of the expression of Lamin B1 after 400 μM $CoCl_2$ treatment. (f) Real-time PCR analysis of relative NLRP1 and NLRC4 mRNA expression in C2C12 myoblasts treated with or without 400 μM $CoCl_2$ for 24 hours. (g) Quantification of NLRP3 protein expression (n=3).The data are shown as the mean ± SD. *, $p<.05$; ***, $p<.001$; NS, no significant difference.

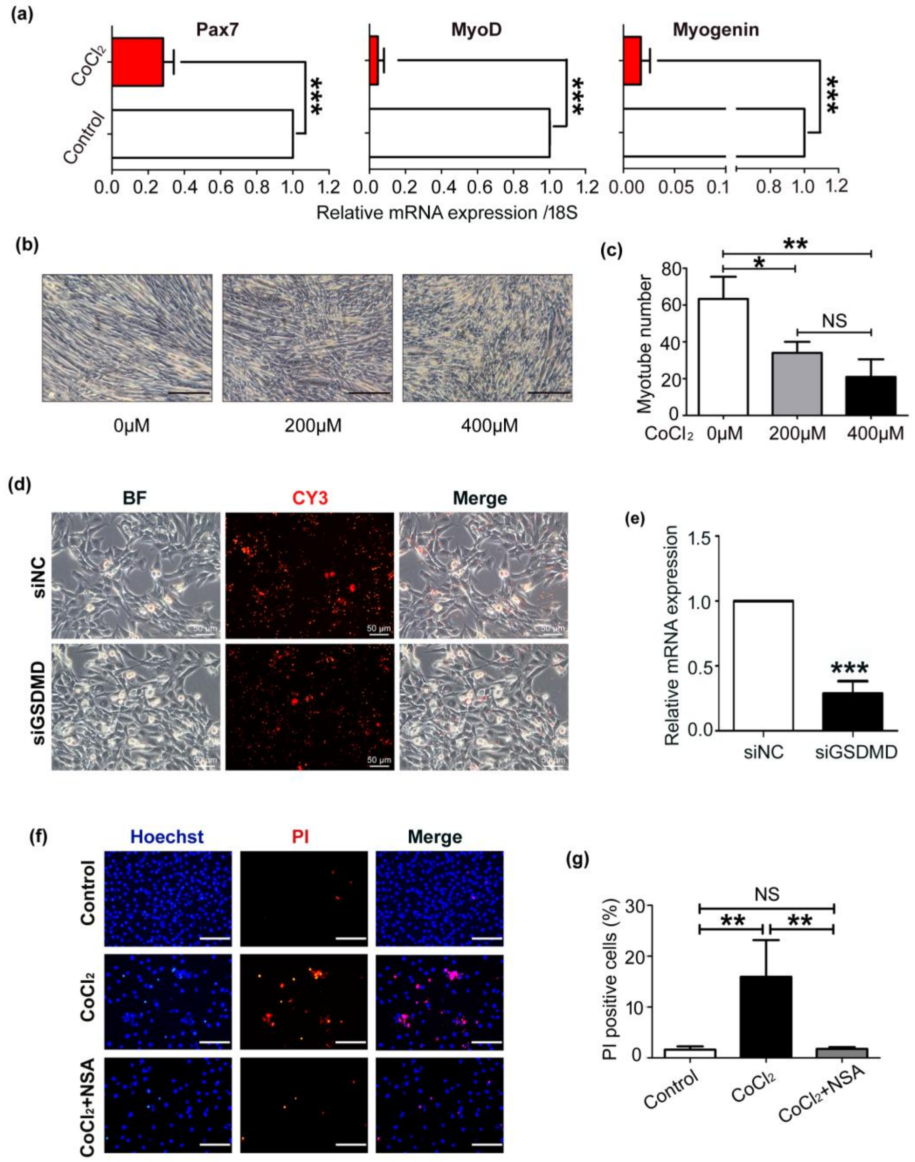

FIGURE S 2: (a) Real-time PCR analysis of relative Pax7, MoyD and Myogenin mRNA expression in C2C12 myoblasts treated with or without 400 μM $CoCl_2$ for 24 hours. (b)-(c) Hypoxia inhibited myotube formation. C2C12 cells underwent myogenic differentiation for 5 days and were then treated with $CoCl_2$ for 48 hours. (d)-(e) C2C12 cells after transfection with siNC or siGSDMD. NC indicates negative control. (f)-(g) Hoechst/PI double staining of C2C12 cells. A GSDMD inhibitor (NSA) partly inhibited hypoxia-induced C2C12 cell death. Scale bars= 50 μm.

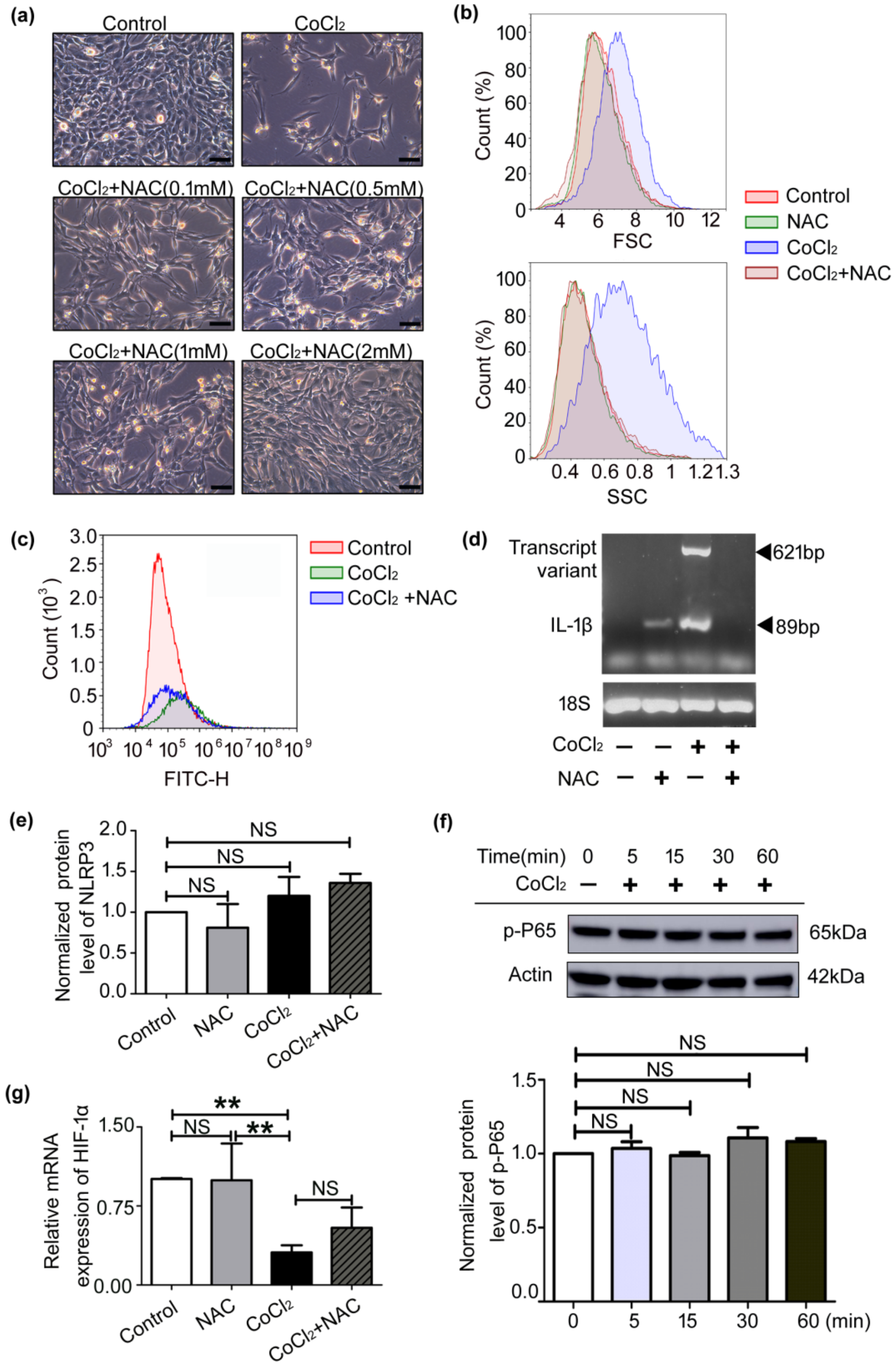

FIGURE S3: (a) Effects of NAC treatment (0-2 mM) followed by $CoCl_2$ treatment in C2C12 cells for 24 hours. At 2 mM, NAC remarkably protected against cell death evoked by $CoCl_2$ treatment. (b) Flow cytometry analysis of the relative cell size (FSC)

and cell complexity (SSC) of C2C12 cells treated with or without NAC under normoxic control and $CoCl_2$-induced hypoxic conditions for 24 hours. (c) Flow cytometry analysis of ROS levels (FITC channel) in C2C12 cells treated with or without NAC under normoxic control and $CoCl_2$-induced hypoxic conditions for 24 hours. (d) Gel electrophoresis of IL-1β after real-time PCR amplification treated with or without NAC under normoxic control and $CoCl_2$-induced hypoxic conditions for 24 hours. (e) Quantitative analysis of NLRP3 protein expression in C2C12 cells treated with or without NAC under normoxic control and $CoCl_2$-induced hypoxic conditions for 24 hours. (f) $CoCl_2$ treatment did not affect the expression level of phosphorylated NF-κB P65 in C2C12 cells within 60 minutes. (g) Real-time PCR analysis of relative HIF-1α mRNA expression upon treatment with or without NAC (n=3). The data are shown as the mean ± SD. **, $p<.01$; NS, no significant difference.
